# A Central Nervous System Disease of Unknown Cause That Occurred in the Minamata Region: Results of an Epidemiological Study

**DOI:** 10.2188/jea.JE20190173

**Published:** 2020-01-05

**Authors:** Shoji Kitamura, Chuzo Miyata, Minoru Tomita, Shoki Date, Terukazu Kojima, Hirosi Minamoto, Susumu Kurimoto, Yoshiyuki Noguchi, Ryoji Nakagawa

**Affiliations:** Department of Public Health, Kumamoto University School of Medicine, Kumamoto, Japan

Recently, a series of cases of a central nervous system disease of unknown cause with extrapyramidal tract abnormalities as the most prominent feature occurred, mainly in fishermen’s communities, on the periphery of Minamata City. Because the symptoms of the disease were unique and severe, and its prognosis was extremely poor, the disease caught immediate attention.

In response to the request from the local Minamata Strange Disease Countermeasures Committee to investigate this disease, we visited this area many times since September 1956 and conducted a detailed epidemiological study, including face-to-face interviews of 40 households with patients and 68 adjacent households without patients as control households. The results of the study are described below.

## GEOGRAPHICAL AND METEOROLOGICAL CONDITIONS OF THE AREA WHERE CASES OCCURRED AND LIVING CONDITIONS OF LOCAL RESIDENTS

**Figure 1.**
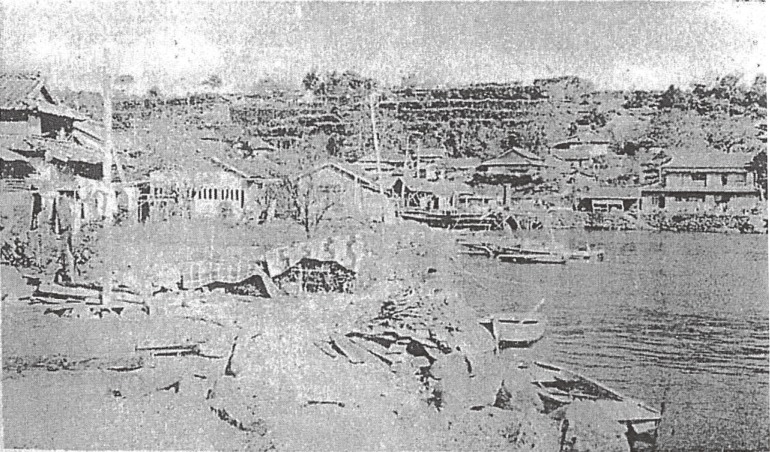
Yudo community

**Figure 2.**
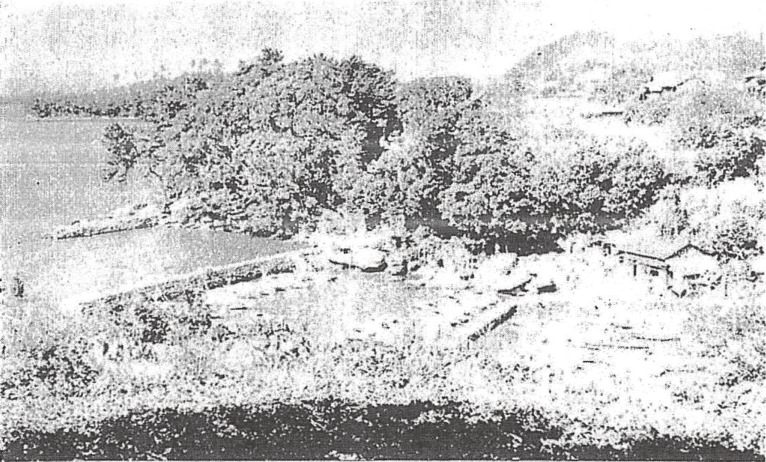
Tsukinoura community

**Figure 3.**
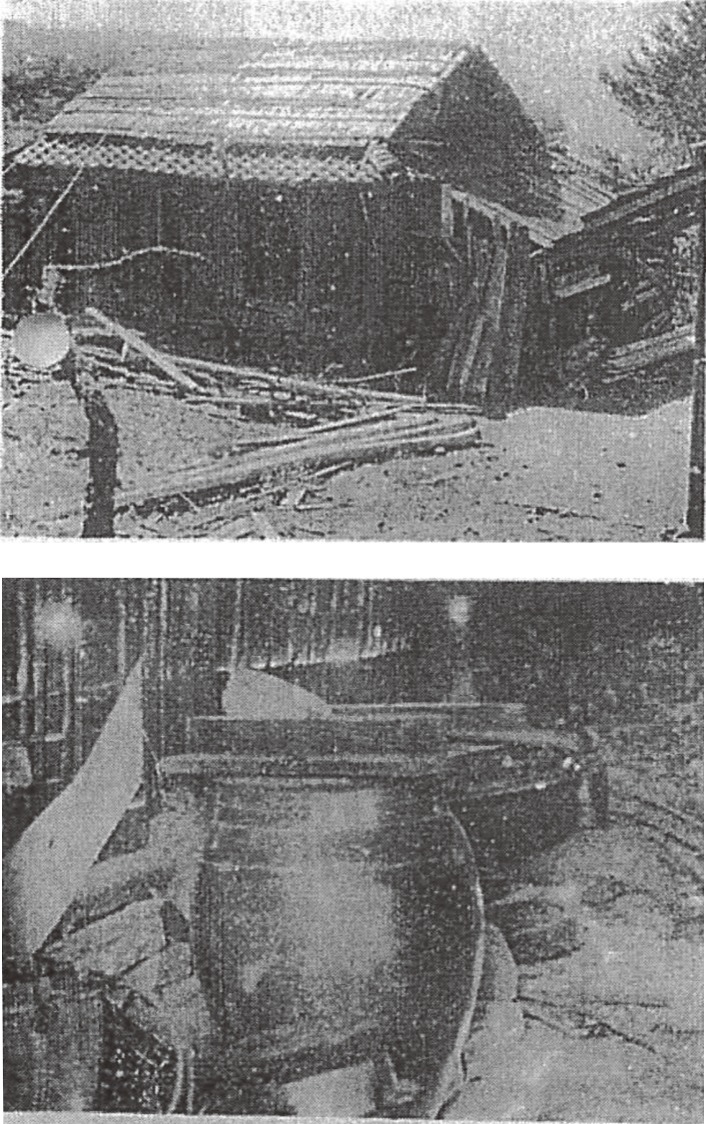
An example of a house where a case occurred. The kitchen of the house above.

**Figure 4.**
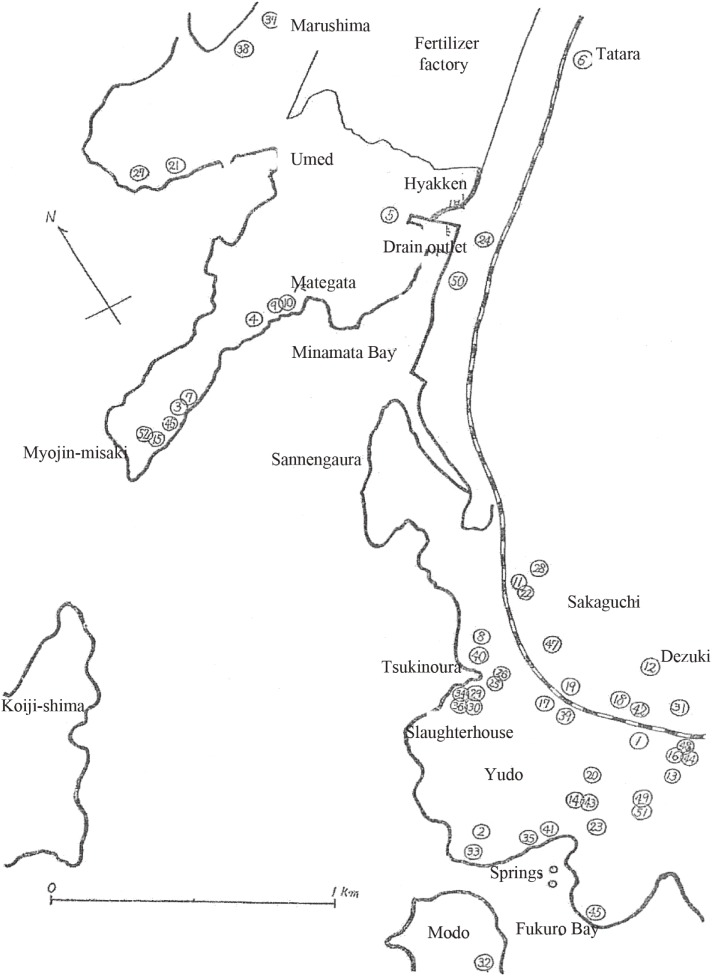
Distribution of cases

The cases occurred at the periphery of Minamata City, which is located in the southernmost part of Kumamoto Prefecture, a scenic waterfront and bay district neighboring Hyakken Port. In particular, a large number of cases occurred in four communities: Myojin, Tsukinoura, Dezuki, and Yudo (see Figure 1, Figure 2, Figure 3, and Figure 4).

These communities are fishing villages located in strips stretching from the seashore to relatively steep hills. Many villagers engage in fishing in the sea and in the bay. Their standards of living are low. Their living environments, including housing, are substandard and unhygienic (see Figure 3). The fresh water supply is poor. Their dietary staples are rationed rice, wheat, and sweet potatoes. They raise some of their own wheat and sweet potatoes. Besides these staples, they consume a large amount of seafood caught locally, while their intake of vegetables and fruits is low.

The meteorological conditions of this area according to the Minamata City handbook (1956) are shown in Table 1. The area is warm and rainy. Its predominant wind direction is northwest. There are no other noteworthy findings.

**Table 1.** Weather in Minamata City (from the 1956 Minamata City handbook)

	1954							1955				
	June	July	August	September	October	November	December	January	February	March	April	May
Temperature, °C												
Mean	22.6	26.6	27.3	24.4	18.8	14.2	9.6	6.8	7.5	9.7	13.9	18.8
Maximum	30.1	32.4	35.2	32.3	27.1	24.8	21.2	13.9	20.0	26.1	23.6	26.8
Minimum	11.0	19.9	21.7	12.0	6.8	6.5	0.6	−2.5	−3.6	2.0	2.4	10.5
Mean humidity, %	77	90	73	30	64	52	57	58	60	64	66	69
Rainfall, mm												
Total	756.3	747.8	255.2	327.0	61.4	40.4	21.0	73.9	113.1	183.0	345.3	153.4
Maximum daily	140.7	98.7	164.5	132.6	27.7	19.6	4.5	34.8	38.3	22.4	129.8	17.4
Number of days												
Clear	7	5	27	17	20	18	19	11	16	10	14	12
Cloudy	15	19	2	9	8	10	10	15	8	13	9	14
Rainy	8	7	2	4	3	2	2	3	3	8	7	5
Snowy	0	0	0	0	0	0	0	2	1	0	0	0
Most frequent wind direction	NW	SE	NW	NW	E	SE	N	NW	SE	NW	NW	NW
Mean wind speed, m/s	2.1	3.2	4.6	2.4	2.2	1.9	2.3	2.7	2.8	2.5	3.2	3.1
Maximum wind speed, m/s	7.0	6.5	7.0	5.5	4.8	7.5	6.0	7.0	7.5	5.5	6.0	5.5

## NUMBER OF CASES THAT OCCURRED BY YEAR AND MONTH

The number of patients with this disease identified based on interviews with local hospitals and clinics is shown chronologically in Table 2.

**Table 2.** Number of cases by month and year

	1953	1954	1955	1956	Total
January				1	1
February					
March				2	2
April		2	1	3	6
May		2	1	5	8
June		2	1	8	11
July			2	2	4
August		4		1	5
September		1	1	5	7
October			1	1	2
November		2	1	2	5
December	1				1

Total	1	13	8	30	52

Confirmed cases of this disease first occurred at the end of 1953 and, as shown in the table, increased suddenly in 1956. The number of patients by month shows marked seasonal variation. The number is high in the period from April to September and low in winter.

## DISTRIBUTION OF PATIENTS BY SEX AND AGE

There are no unusual findings regarding the population composition by age and sex (Table 3). Cases have generally occurred irrespective of sex or age, except that cases have tended to occur a little more frequently in children aged 10 years or younger. The youngest patient is a girl who was 1 year and 10 months of age at onset. There have been no infant cases, although diagnosis of this disease in infants is difficult.

**Table 3.** Distribution of patients by sex and age

Age	≤9 y		10–19 y	20–29 y	30–39 y	40–49 y	50–59 y	60–69 y	70–79 y	Total
	≤4	≥5								
Males	4	6	3	3	4	7	3	1	0	31
Females	6	4	4	2	0	3	2	0	0	21

Total	10	10	7	5	4	10	5	1	0	52

## INCIDENCE PROPORTION, CASE FATALITY RATE, AND PROGNOSIS OF THIS DISEASE

The total population of these districts is 10,119. The 3-year incidence rate up to the present is 51.3 per 10,000 people. When exclusively examining fishermen’s communities by excluding the Marushima, Hyakken, and Tatara districts, the incidence proportion doubles to 102 per 10,000 people (Table 4).

**Table 4.** Number of households, population, and number of cases that occurred by district

District	Number of households	Population	Number of patients
Tsukinoura	100	498	16
Yudo	121	649	11
Dezuki	96	453	7
Myojin	104	491	5
Mategata	70	343	3
Hyakken	243	1,192	3
Umedo	172	978	2
Marushima	775	3,839	2
Tatara	125	586	1
Sakaguchi	94	513	1
Modo	119	577	1

Seventeen patients have died from this disease. The case fatality rate is extremely high at 32.8%. Time from onset to death is, as shown in Table 5, as short as 20 days and as long as 2 years and 3 months. Only 3 of the 52 patients were able to return to work, and still have sequelae. There are 23 patients who remain bedridden after onset. The amount of time for which they have been confined to bed up to the present is, as shown in Table 5, 1 year or longer for 6 patients. One of these patients has been bedridden for 2 years and 3 months. Prognosis is extremely poor.

**Table 5.1.** Patient outcomes

At work	Improved	Not improved	Dead	Total
3	9	23	17	52

**Table 5.2.** Time from onset to death

0–1 month	≤2 months	≤6 months	≤1 y	≤1 y and 6 months	≤2 y	2–3 y	Total
2	6	3	4	1	0	1	17

**Table 5.3.** Time of confinement to bed up to present in patients who have not improved

0–6 months	7–12 months	≤2 y	≤3 y	Total
13	4	5	1	23

## GEOGRAPHICAL DISTRIBUTION, ORDER OF ONSET, AND FAMILIAL AGGREGATION OF PATIENTS

Figure 4 is a sketch of a map of the area, with sites where cases occurred plotted using numbers in the order of occurrence (the seashore district to the east of Sannengaura in the figure, where no cases have occurred, has no houses). As shown in Table 6, relatively more cases occurred in the Tsukinoura and Yudo districts in 1956 (site number 23 and higher). However, overall, there is no tendency for the distribution of cases to gradually expand or move. Intervals between the dates of onset within the same household, as shown in Table 7, vary widely from as short as 6 days to as long as 1 year and 5 months. Based on these observations, it is highly unlikely that this disease was transmitted through a chain of infection.

**Table 6.** Number of cases that occurred by district and year

	Tsukinoura	Sakaguchi	Yudo	Dezuki	Modo	Myojin	Hyakken	Mategata	Umedo	Marushima	Tatara	Total
1953	0	0	0	1	0	0	0	0	0	0	0	1
1954	2	0	2	2	0	2	1	3	0	0	1	13
1955	1	0	1	4	0	1	0	0	1	0	0	8
1956	8	1	8	5	1	2	2	0	1	2	0	30

Total	11	1	11	12	1	5	3	3	2	2	1	52

**Table 7.** Intervals between the dates of onset within the same family

District	Relationship with the householder and age of onset	Date of onset	Interval between the dates of onset
Myojin	Eldest son	20 June 1955	1 year, 4 months, and 20 days
	Householder	10 November 1956	

	Eldest son	25 April 1954	1 month and 15 days
	Householder	10 June 1954	

Mategata	Eldest daughter	10 August 1954	6 days
	Householder	16 August 1954	

Yudo	Eldest daughter of the eldest son	24 September 1956	2 months and 18 days
	Eldest son of the eldest son	12 November 1956	

	Third son	26 May 1955	1 year, 1 month, and 19 days
	Eldest daughter	14 July 1956	

Dezuki	Third son	September 1955	Approximately 1 year 7 days
	Householder	15 September 1956	
	Wife	22 September 1956	

Tsukinoura	Nephew	27 August 1954	1 year, 2 months, and 20 days
	Younger brother	15 November 1955	

	Fourth daughter	End of April 1956	Approximately 2 weeks Approximately 2 weeks Approximately 2 weeks
	Fourth son	May 1956	
	Wife	26 May 1956	
	Fifth son	June 1956	

Editorial note: the original paper had the column showing “name of householder” in this table, but the editor of the Journal of Epidemiology omitted it to prevent potential ethical issues.

The familial aggregation rate (the proportion of patients from families with at least two patients) is 21/52, that is, approximately 40%, which is much higher than that for other infectious central nervous system diseases.

While most patients are permanent residents in this area, two patients developed the disease 6 months after migrating from remote areas.

## DISTRIBUTION OF THE OCCUPATIONS OF HOUSEHOLDS WITH PATIENTS

Table 8 compares the occupations of householders between households with patients and control households. Although it is expected that this area would contain a large number of fishermen’s households, there was a marked difference in the proportion of fishermen’s households between households with patients and control households. A finding that was particularly noteworthy was that of the 14 non-fishermen’s households with patients, 10 households contained at least one person, either the householder or a family member, who engaged in fishing in one way or another, while the remaining 4 households did not contain anyone who engaged in fishing but are adjacent to fishermen’s households and could obtain seafood caught locally. No similar findings were found in the control households without patients.

**Table 8.1.** Occupations of households with patients and control households

	Fishing	Farming and fishing	Farming	Other	Total
Households with patients	22 (55%)	4 (10%)	2 (5%)	12 (30%)	40
Control households	10 (14.7%)	3 (4.4%)	15 (22.1%)	40 (58.8%)	68

**Table 8.2.** Households with and without patients according to employment in fishing

	Households with patients	Control households
Employment in fishing	36 (90%)	20 (29.4%)
No employment in fishing	4 (10%)	48 (70.6%)

Total	40	68

The single case in the Tatara district, which occurred away from the seashore (number 6 in Figure 4), is an office worker who enthusiastically engaged in fishing. A patient in Hyakken (number 50 in Figure 4) is a barber but engaged in fishing for one-third of a month. These examples explain the circumstances by which cases occurred.

## DEATH OF DOMESTIC ANIMALS RAISED LOCALLY

A fact that is related to the development of this disease and is extremely specific to this disease is that many cats, and domestic animals raised locally, developed symptoms similar to those of patients and died.

Deaths of cats by district and year confirmed through interviews are shown in Table 9. Most cats that were raised in households with patients have died. The cats generally died 1–2 months before the case deaths in the same household. The number of cat deaths by year was 1, 18, 25, and 30 since 1953, demonstrating a similar pattern to the increase in the number of human patients by year.

**Table 9.** Number of cat deaths

	Tsukinoura	Dezuki	Yudo	Myojin	Mategata	Hyakken	Umedo	Marushima	Tatara	Total
Households with patients (40)										
Number of cats raised	14	15	18	4	4	2	3		1	61
Number of deaths	13	10	15	4	4	1	2		1	50
1953										0
1954	3		6	1	3		1		1	15
1955	4	5	3	2	1					15
1956	6	5	6	1		1	1			20
Control households (68)										
Number of cats raised	12	23	13	2	1	2	3	2	2	60
Number of deaths	3	5	10	2	1	1	2			24
1953		1								1
1954		1		1	1					3
1955	2	2	4			1	1			10
1956	1	1	6	1			1			10

The number of deaths of dogs and domestic animals is shown in Table 10. The circumstances of these deaths are not clear. Among these, however, 5 pigs and 1 dog ingested seafood, developed symptoms similar to those of cats, and died, according to the interviews.

**Table 10.** Status of raised domestic animals

	Households with patient(s)		Control households	
	Number of domestic animals currently being raised	Number of deaths in recent years	Number of domestic animals currently being raised	Number of deaths in recent years
Rabbit	8	0	29	2
Dog	10	3	6	1
Goat	3	1	3	1
Horse	1	0	1	0
Cattle	0	0	3	0
Pig	26	5	53	3
Chicken	85	2	313	0

Based on the results of bacteriological and virological examinations and clinical and pathological findings, this disease is slightly different from known infectious or familial/hereditary central nervous system diseases. As described above, the distribution of cases did not expand or move chronologically or geographically, and intervals between the dates of onset within the same household varied widely. These findings suggest that a person-to-person chain of infection is unlikely. Moreover, considering that the familial aggregation rate is extremely high, the complete cure rate is equal to 0, the disease course can be very long, the prognosis is very poor, and cases have occurred irrespective of the sex or age of patients, conventional infectious central nervous system diseases, including Japanese encephalitis, epidemic meningitis, and acute poliomyelitis, can be ruled out.

Pathological and clinical findings suggest that this disease is caused by a toxic substance or substances rather than an infectious disease. Epidemiological findings show that cases occurred sporadically in space and continuously in time. It is also impossible to identify a starting point from which the disease developed for each patient. Therefore, if the disease is the result of poisoning, it develops after long-term continuous exposure to a common cause specific to these districts.

Deaths of domestic cats may suggest that cats are an animal source of infection to humans. However, it can also suggest that both cats and humans were exposed to a common cause and developed poisoning. The results of the search for this common cause are described below.

## INTAKE OF DRINKING WATER AND FARM PRODUCE

Residents of this area generally drink well water. Because the water supply is limited, quite a few households use wells that are shared among many other households. The usage of wells is shown in Table 11. Table 12 shows that, even among households using the same shared well, there are households with patients and without patients. Moreover, as shown in Table 11, four cases occurred in households that used the Minamata City’s water supply. This clearly demonstrates that contamination of drinking water is not the cause of this disease.

**Table 11.1.** Drinking water

	Water source				

	Well				Tap water

	Shared use	Exclusive use	Open	Closed	
Households with patients (40)	28	10	36	2	4
Control households (68)	44	17	55	6	7

**Table 11.2.** Water consumption (daily per person)

	0–0.9 “to”	1–1.9 “to”	2–2.9 “to”	3–3.9 “to”	≥4 “to”
Households with patients (40)	16	13	5	2	1
Control households (68)	19	29	5	6	2

Traditional unit of volume, 1 to = approximately 18 liters.

**Table 12.** Households with patients and without patients using shared wells

Shared well	A	B	C	D	E	F
Number of households using a shared well	12	4	6	6	6	9
Number of households with patients	3	2	2	1	1	1
(number of patients)	(3)	(4)	(2)	(1)	(1)	(1)
Number of households without patients	9	2	4	5	5	8

This area contains acid soil composed of pyroxene andesite and is a narrow plain along the seashore. Therefore, even farmers’ households have limited farm produce. Almost no rice is cultivated. Many people purchase rationed rice for consumption. Self-sufficient use of wheat and sweet potato as substitutes for the staple is not different between households with patients and those without patients (Table 13). Households with patients in Tatara and Hyakken purchase farm produce from the Minamata market. Based on these facts, farm produce is not worth considering as a potential source of contamination. Rather, the poorer conditions of households with patients, which can be presumed by the number of tatami mats per person (Table 14), along with no or little area for dry field farming indicate that households with patients may have imbalanced nutrient intake or a lack of vitamins and minerals.

**Table 13.** Self-sufficiency of staples

	Self-sufficient	Partially self-sufficient	Purchasing all staples	No access to rationed food
Rice				
Households with patients (40)	1 (2.5%)	1 (2.5%)	36 (90%)	2 (5.0%)
Control households (68)	3 (4.4%)	0 (0%)	59 (86.8%)	6 (8.8%)
Wheat/Sweet potato				
Households with patients (40)	13 (32.5%)	12 (30.0%)	15 (37.5%)	—
Control households (68)	22 (32.3%)	19 (27.9%)	27 (39.8%)	—

Land under cultivation		None	≤1 “tan”	≤2 “tan”	≤5 “tan”	>5 “tan”
Rice field						
	Households with patients (40)	38	0	1	1	0
	Control households (68)	65	0	1	2	0
Dry field						
	Households with patients (40)	13	12	6	8	1
	Control households (68)	18	24	14	10	2

Traditional unit of area, 1 tan = approximately 1000 m^2^.

**Table 14.** Number of tatami mats per person

	0–0.9	1.0–1.9	2.0–2.9	3.0–3.9	4.0–4.9	5.0–5.9	≥6.0	Total
Households with patients	8	12	11	4	4	1	0	40
Control households	4	14	20	16	9	4	1	68

## INTAKE OF SEAFOOD AND FISHING METHODS

Many local residents are fishermen and, as expected, consume a large amount of seafood. Compared to other areas, this area is unique in that the residents mainly consume seafood caught in the bay. There are similar fishing villages on the seashore immediately northeast and southwest of this area. However, no cases occurred in these communities, and fishermen in these communities do not fish in Minamata Bay because their fishing zones are different. In contrast, fishermen’s households in this area, from Marushima to Modo, engage in fishing mainly in this bay.

In general, most fishing methods used by local fishermen are very small in scale and can be broadly divided into gill net fishing, dragnet fishing, pole-and-line fishing, octopus fishing, torch fishing, and shellfish harvesting. Gill net fishing uses a vinyl net and was started in 1952. This method is similar to that using mist nets on land. Usually, a net is set in the sea the previous day and is dragged in the next morning. Fishing grounds are located in Minamata Bay. In particular, the coasts of Myojin and Tsukinoura are good fishing grounds. Fish species caught by gill net fishing include gizzard shad, sillaginoid, spotnape ponyfish, flatfish, white croaker, flathead, redlip mullet, skilfish, black rockfish, marbled rockfish, prawn, crab, and mantis shrimp. Some fishermen engage in gill net fishing all year round, while many fishermen switch to the pole-and-line fishing of striped mullets in the summer. In winter, the frequency of gill net fishing and the catch decreases slightly. In the Yudo district, where the number of cases increased in fiscal year 1956, cases occurred in 5 of the 7 households that engaged in gill net fishing in the same fiscal year. At the end of 1955, these households moved their gill net fishing location from a southern part of the bay, in the vicinity of Fukuro Bay, to the northern parts, Myojin and Tsukinoura.

Fishing grounds for dragnet fishing are Fukuro Bay and the seashore of Tsukinoura. The fishing season is year-round, but the frequency of dragnet fishing is low. The main catch is Japanese anchovies. Sometimes gizzard shads and other small fish are also caught.

Fishing grounds for pole-and-line fishing and octopus fishing are in the bay. The main catch by pole-and-line fishing is striped mullets. Other fish caught are lizardfish and cutlassfish. Catching striped mullets is the main contribution to the high catch of fish in summer.

Torch fishing, also called harpooning, is mainly used to catch sea cucumbers, abalones, octopuses, marbled rockfish, and others using carbide lamps at night on the coast. Fishing grounds comprise the entire coast of the bay and the fishing season is year-round.

Shellfish, specifically oysters, small snails, and Manila clams, are mainly harvested from November to March. Because of the tidal rhythm, they are caught for a week per month. In recent years, seaweed has not been harvested in the bay.

Comparison of these fishing methods between households with patients and control households is shown in Table 15. Even after accounting for the difference in the number of fishermen’s households, the proportion of households that engaged in gill net fishing, octopus fishing, torch fishing, and shellfish harvesting was larger among households with patients than control households.

**Table 15.** Fishing methods

	Households with patients (40)		Control households (68)	
	Number of households using the method	%	Number of households using the method	%
Gill net fishing	15	37.5	6	8.8
Dragnet fishing	2	5.0	2	2.9
Pole-and-line fishing	20	50.0	19	28.0
Octopus fishing	8	20.0	2	2.9
Torch fishing	8	20.0	2	2.9
Shellfish/oyster harvesting	16	40.0	6	8.8

We identified the seafood species ingested by season through interviews and found marked differences between households with patients and control households (Table 16). That is, patients predominantly consumed fish caught using the methods described above in the bay, while most control households consumed fish caught outside the bay; specifically, horse mackerel, mackerel, sardines, sea bream, and others purchased from the Minamata market or through peddlers from Komenotsu, Kagoshima Prefecture. The seafood classification and intake, which were investigated by identifying the frequency of consumption per month, are shown in Table 17 and demonstrate that households with patients consumed a very large amount of fish and shellfish from the bay.

**Table 16.** Seafood ingested

Species	Fishing		Households with patients (40)					Control households (68)				
	Location	Method	Spring	Summer	Fall	Winter	Total	Spring	Summer	Fall	Winter	Total
Lizardfish	In the bay	Gill net fishing, Fishing	3	4	2	0	9	2	0	1	1	4
Gizzard shad	〃	Gill net fishing	20	10	16	12	58	9	6	3	4	22
Sillaginoid	〃	Fishing	10	7	10	8	35	4	3	4	1	12
Spotnape ponyfish	〃	Gill net fishing	10	3	6	6	25	4	3	1	1	9
Flatfish	〃	Gill net fishing	9	3	6	7	25	2	3	5	1	11
White croaker	〃	Gill net fishing	9	2	9	6	26	2	1	2	1	6
Flathead	〃	Gill net fishing	10	4	9	6	29	1	0	1	1	3
Redlip mullet	〃	Gill net fishing	11	3	8	9	31	1	0	1	1	3
Skilfish	〃	Gill net fishing	6	0	6	9	21	4	3	4	3	14
Black rockfish	〃	Gill net fishing	2	0	0	1	3	0	0	0	0	0
Marbled rockfish	〃	Torch fishing	3	1	0	1	5	0	1	0	0	1
Striped mullet	〃	Fishing	8	24	21	2	55	5	23	22	1	51
Cutlassfish	〃	Fishing	4	14	16	5	39	6	13	18	6	43
Black sea bream	〃	Fishing	2	4	2	1	9	2	1	0	2	5
Japanese anchovy	〃	Dragnet fishing	2	3	3	2	10	4	2	1	3	10
Puffer fish	〃	Fishing	0	0	0	1	1	0	0	0	0	0
Prawn	〃	Gill net fishing	10	1	8	7	26	4	4	3	0	11
Crab	〃	Gill net fishing	16	11	12	9	48	4	4	3	2	13
Mantis shrimp	〃	Torch fishing	7	0	8	6	21	1	0	1	1	3
Sea cucumber	〃	Torch fishing	3	0	0	8	11	1	0	0	1	2
Octopus	〃	Torch fishing, Octopus fishing	9	13	2	4	28	6	8	6	2	22
Squid	Outside the bay		3	4	1	2	10	3	0	1	3	7
Oyster	In the bay	Shellfish harvesting	17	3	1	26	47	8	1	2	23	34
Small snails	〃	Shellfish harvesting	13	10	5	13	41	11	4	2	9	26
Manila clam	〃	Shellfish harvesting	5	2	3	2	12	6	2	2	4	14
Abalone	〃	Torch fishing	2	0	0	4	6	1	0	1	1	3
Horse mackerel	Outside the bay		16	11	18	12	57	35	28	39	45	147
Mackerel	〃		15	6	12	11	44	29	19	37	35	120
Sardine	〃		17	7	14	14	52	46	30	36	39	151
Saury	〃		0	0	1	0	1	0	0	2	0	2
Silver-stripe round herring	〃		1	0	1	1	3	2	3	2	2	9
Sea bream	〃		0	2	1	0	3	1	3	2	0	6
Wakame seaweed	〃		0	0	0	1	1	0	0	0	0	0

**Table 17.** Intake of seafood

	Bay fish	Oysters and other shellfish	Non-bay fish
Households with patients (40)			
Eat almost every day	25	5	2
Eat 2–3 times a week	10	11	2
Eat 2–3 times a month	3	6	6
Control households (68)			
Eat almost every day	4	5	12
Eat 2–3 times a week	19	12	25
Eat 2–3 times a month	23	12	24

The species of fish and shellfish from the bay that were consumed in large amounts included gizzard shad, striped mullet, crabs, oysters, and small snails. No particular type of fish or shellfish was commonly consumed in large quantities by patients. There was no association between the fish and shellfish consumed and development of the disease. The fish and shellfish were either eaten raw or cooked; there was no preference in cooking method. Some of the patients had never eaten raw seafood. In addition, pigs, which usually do not eat raw food, also died. The fact that development of the disease is not associated with any particular fish or shellfish, or the consumption of raw fish or shellfish may rule out the possibility that this disease is caused by a biological toxin present in specific fish or shellfish or that it is a parasitic disease mediated by fish or shellfish, even if ingestion of fish and shellfish from the bay is the cause of this disease.

A noteworthy fact about the species of fish and shellfish inhabiting the bay is that a few of these species contain aneurinase and were consumed in relatively large quantities.

**Figure 5.**
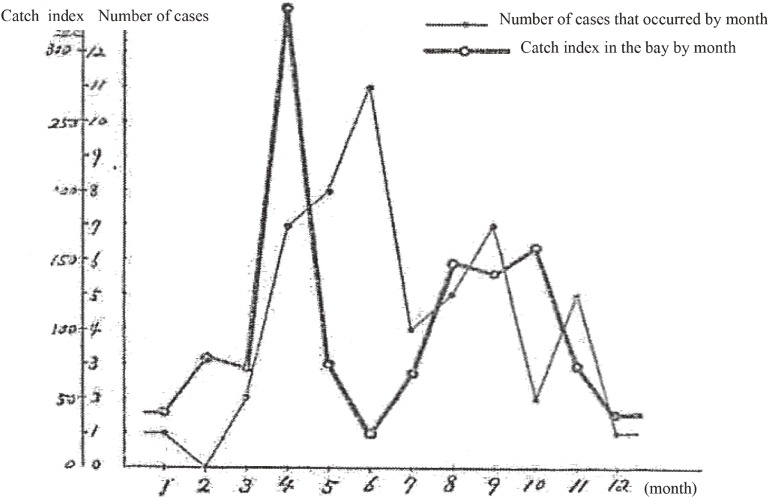
Relationship between the catch index in the bay and the number of cases that occurred by month

Table 18 shows the change in catch of fish in the bay by month in a year for households with patients, which was investigated at the fishermen’s union office in Marushima. The catch of fish decreases in the rainy season and in winter. When this change is compared with the change in the number of cases that occurred by month (Figure 5), the two changes appear similar with a slight lag.

**Table 18.** Catch of fish in the bay by month

1955	Catch of fish (“kan”)	Index compared to the monthly mean catch (100)	Proportion of the annual catch (%)
January	48	42.1	3.4
February	91	79.7	6.6
March	80	70.2	5.8
April	376	330.0	27.4
May	86	75.5	6.3
June	30	26.3	2.2
July	77	67.3	5.6
August	164	144.0	12.0
September	160	140.0	11.7
October	127	111.0	9.3
November	86	75.5	6.3
December	47	41.2	3.4

Traditional unit of weight, 1 kan = approximately 3.75 kg.

The results described above indicate that, if this disease is a poisoning, fish and shellfish in the bay are contaminated for some reason, and relatively long-term ingestion of these fish by local residents and domestic animals leads to development of this disease. That is, the common cause for entry of a toxic substance into the body is conceivably fish and shellfish from the bay. This explains the seasonal change in the development of the disease, the absence of the disease in infants, and the predominance of cats among the dead pets and domestic animals.

However, if this is the case, the details of individual seafood intake suggest that the predisposition of an individual plays a significant role in the development of this disease. If the fish and shellfish in this bay are contaminated, it is unknown why the contamination started near the end of 1953. Lastly, we will present the results of our investigation into special environments that may have caused the contamination.

## SPECIAL ENVIRONMENTS OF THE AREA WHERE CASES OCCURRED

Special environments in the area where cases occurred that could have contaminated the bay are the Minamata factory of a fertilizer corporation, a municipal slaughterhouse in the Tsukinoura district, springs in the sea in the Yudo district (shown in Figure 4), and the former Japanese navy ammunition depot and high-angle gun position in the Modo district.

Wastewater from the fertilizer factory is released into the Hyakken Port entrance. Analytical values of inorganic salts contained in the wastewater (measured by the engineering department of the factory) are shown in Table 19. Toxic gases contained in exhaust gas from the factory are sulfurous acid gas and nitrogen oxide, which are usually produced at sulfuric acid plants.

**Table 19.** Analytical values of wastewater from a fertilizer factory

pH	3.5	
Total residue on evaporation	9,900	mg/L
KMnO_4_ consumption	155	
SiO_2_	23	
FeCl_3_	2	
Al_2_O_3_	19	
CaO	163	
MgO	436	
K_2_O	114	
Na_2_O	2,700	
NH_3_	24	
Cu	5	
Pb	0.13	
As	0.001	
Mn	0.17	
Cl	3,950	
P_2_O_5_	9	
SO_3_	676	

The slaughterhouse is located at the top of a hillock facing the Tsukinoura coast, and its wastewater is released into the sea. The number of domestic animals slaughtered in recent years is shown in Table 20.

**Table 20.** The number of domestic animals slaughtered recently in Minamata City

Fiscal year	Species					Remarks
	Cattle	Pig	Horse	Goat	Total	
1946	211	62	40	0	313	April–March in every fiscal year
1947	207	26	14	0	247	〃
1948	294	41	14	0	349	〃
1949	178	166	8	0	352	〃
1950	364	167	18	0	549	〃
1951	268	266	9	10	553	〃
1952	323	975	6	0	1,304	〃
1953	362	758	129	0	1,249	〃
1954	608	562	101	0	1,271	〃
1955	1,554	643	30	0	2,227	〃
1956	984	555	31	0	1,570	In April 1, 1956 to November 30, 1956

Total	5,353	4,221	400	10	9,984	

No changes in the water from the springs in the sea in the Yudo district have been detected in recent years. Similarly, there has been no change in the farming of young sweetfish, which has been performed for some time.

The ammunition stored in the Modo district was removed by stationed troops after World War II and the remaining parts were purchased and transported by ship. These were not dumped into the sea.

## SUMMARY

The results of the epidemiological study on this central nervous system disease of unknown cause occurring in the Minamata region are as follows:

1. Cases started to occur at the end of 1953. The number of patients was 13 in 1954, 8 in 1955, and dramatically increased to 31 in 1956 (as of the end of November), resulting in a total of 52 patients in 3 years.
2. The number of cases that occurred per month was higher in April–September and lower in winter, demonstrating marked seasonal change.
3. Case numbers are similar, irrespective of age and gender. However, there have been no infant cases.
4. The case fatality rate of this disease is 33%. Most patients’ symptoms have not changed for a long time, and no patient has been cured completely. The prognosis of this disease is extremely poor.
5. The area where cases occurred is limited to farmers’ and fishermen’s communities on the seashore of Hyakken Port in Minamata City and is not expanding. In particular, many patients are from fishermen’s households, and the familial aggregation rate is extremely high at 40%. Moreover, many cats raised in the same area had similar symptoms and have died.
6. It has been found that this disease develops after long-term continuous exposure to a common cause, which is conceivably contaminated fish and shellfish inhabiting the bay.

